# *In silico *engineering of aggregation-prone recombinant proteins for substrate recognition by the chaperonin GroEL

**DOI:** 10.1186/1471-2164-13-S7-S22

**Published:** 2012-12-07

**Authors:** Vipul Kumar, Ankita Punetha, Durai Sundar, Tapan K Chaudhuri

**Affiliations:** 1 School of Biological Sciences, Indian Institute of Technology Delhi, Hauz Khas, New Delhi 110016, India; 2 Department of Biochemical Engineering and Biotechnology, Indian Institute of Technology Delhi, Hauz Khas, New Delhi 110016, India; 3 Department of Biotechnology, Indian Institute of Technology Guwahati, Guwahati 781039, India

## Abstract

**Background:**

Molecular chaperones appear to have been evolved to facilitate protein folding in the cell through entrapment of folding intermediates on the interior of a large cavity formed between GroEL and its co-chaperonin GroES. They bind newly synthesized or non-native polypeptides through hydrophobic interactions and prevent their aggregation. Some proteins do not interact with GroEL, hence even though they are aggregation prone, cannot be assisted by GroEL for their folding.

**Results:**

In this study, we have attempted to engineer these non-substrate proteins to convert them as the substrate for GroEL, without compromising on their function. We have used a computational biology approach to generate mutants of the selected proteins by selectively mutating residues in the hydrophobic patch, similar to GroES mobile loop region that are responsible for interaction with GroEL, and compared with the wild counterparts for calculation of their instability and aggregation propensities. The energies of the newly designed mutants were computed through molecular dynamics simulations. We observed increased aggregation propensity of some of the mutants formed after replacing charged amino acid residues with hydrophobic ones in the well defined hydrophobic patch, raising the possibility of their binding ability to GroEL.

**Conclusions:**

The newly generated mutants may provide potential substrates for Chaperonin GroEL, which can be experimentally generated and tested for their tendency of aggregation, interactions with GroEL and the possibility of chaperone-assisted folding to produce functional proteins.

## Background

In cells, the protein folding mechanism occurs with the help of a very important class of proteins known as molecular chaperones, which bind to non-native proteins and prevent their aggregation. The GroEL is one of the thoroughly studied chaperonin found in *Eschericia coli *that functions in presence of its co-chaperonin GroES and provides the paradigm for chaperonin-assisted protein folding [1,2]. The chaperonin GroEL is a large homo-tetradecamer composed of two back-to-back 7-membered rings of 57-kD subunits, with a central channel or cavity [3-5] at either terminus that are involved in binding with non-native polypeptides.

GroEL's co-chaperonin partner GroES is a single, seven-membered ring of 10-kDa subunits [6]. According to the suggested mechanism, GroEL binds the non-native state of a polypeptide to its hydrophobic cavity via multiple hydrophobic contacts. The expected outcome of the current study is to design mutantsresent in central cavity of GroEL. Subsequently ATP and GroES bind to GroEL, forming a cap over the polypeptide containing cavity and simultaneously causing a conformational change in GroEL that sequesters the hydrophobic surfaces and doubles the volume of central channel. This releases the bound polypeptide into the GroEL central cavity where it folds into its native form according to its primary amino acid sequence [5]. Discharge of the protein into the bulk solvent may occur only when ATP and GroES bind to the opposite ring of GroEL, triggering an unfavourable ring-ring interaction that leads to dissociation of the first GroES and release of the folded protein. The polypeptide released in this way, can be in any of the folding states i.e. the native state, a conformation committed to reaching the native state or an uncommitted state that will result in non-native state. This non-native state can again bind to GroEL for another attempt of folding [7].

It is well established that a part of GroES mobile loop sequence, GGIVLTG, that binds with GroEL [5] must possess desired properties for the stable GroEL-GroES complex formation, which has also been proved by crystal structures [4] and nuclear magnetic resonance data [8]. Heptameric GroES is the natural binding partner for GroEL; however, an isolated mobile loop from GroES monomer should not qualify as a good substrate for GroEL because of the presence of 7 such mobile loops as well as a C7 axis of symmetry could cause a perfect fit in GroEL opening. GroEL preferably binds with polypeptides having multiple hydrophobic patches [9] and hence those polypeptides would behave like its natural substrate.

To uncover the basis for various substrate-protein recognition by chaperonin GroEL, few studies have been carried out in the past involving several *in vivo *and *in vitro *substrates [10]. Some of the basic aspects in the GroEL substrate recognition have been reported from the structural correlation method using local and global hydrophobicity profile of the substrates. In this approach, the local hydropathy index of the specific GroES mobile loop region, GGIVLTG, which is responsible for binding with GroEL, has been considered as standard. The hydropathy indexes of other amino acid sequences were calculated and compared with the standard value and some predictions were made for their potentiality to bind with GroEL [9].

From the above predictions, it is evident that the presence of a mobile loop (GGIVLTG)-type structure in a protein substrate, is an important factor that will determine the favoured interactions of GroEL with that particular substrate. Also the Grand Average Hydropathicity (GRAVY: sum of hydropathy index of amino acid in a sequence divided by the number of amino acids) value of this patch is so high that it can itself provide a site for strong interactions [11]. In the present work, we have reported two proteins that do not have propensity of binding with GroEL, but some of their mutants were shown to be potential substrates for GroEL. For these mutants and their wild type counter parts, energy calculations for the comparison of their relative stability, aggregation propensity and solubility were performed. Based on these parameters as well as on the basis of calculated energy value derived from Molecular Dynamics Simulations [12], the relative stability of the mutants with respect to their wild type counterparts can be predicted.

The expected outcome of the current study may help to design mutants for non-"GroEL binding" aggregation prone proteins, that could potentially bind to GroEL and may be assisted for their correct folding in the *Eschericia coli *cells.

## Methods

### Finding the hydrophobic patch and generating mutants

In this work, we considered proteins that were identified as poor substrate for GroEL in our previous study [9]. A bonafide list was obtained with a number of proteins having poor binding tendency towards GroEL. The structure of most of the proteins in the list of GroEL substrates have been solved through crystallography or NMR spectroscopy, and various parameters related to their stabilization, folding and over-expression are available in the literature. Consequently, we shortlisted important proteins based on the availability of their X-ray crystal structure and other parameters (e.g. temperature for expression) sufficient to mimic the experimental conditions computationally. The selection of the proteins based on the availability of the data, confines the number of shortlisted proteins to two, i.e. Ureidoglycolate hydrolase [13] and Hsp31 protein [14] both found in *E.coli*. The two proteins are potentially convertible to GroEL substrates, whose amino acid sequences were collected from SwissProt Databank and structures from PDB. Here, we intended to develop a hydrophobic patch, or mobile loop region, which is similar to the patch in GroES, and have GRAVY value comparable to that of GGIVLTG for making it a better substrate for GroEL. Hydrophobic amino acid patches in the selected protein candidates, which had high similarity with the GGIVLTG patch, were found using SIM Alignment tool to get the most correlated regions with their correlation values. The patches were chosen to make mutations so that the GRAVY values can approach closer to that of GGIVLTG patch [15] (Table 1). The change in GRAVY values due to single mutations were not considered and double mutants were created for the suggested patches by mutating the charged amino acid residues to hydrophobic residues (preferably I, V or L). The GRAVY values were calculated for the obtained patches using Protparam Tool from Expasy [16] (Table 2).

**Table 1:** Result of SIM Alignment Tool: Hydrophobic patches similar to "GGIVLTG"

**S. No**.	Swissprot ID's	Patch obtained	%age Correlation	Suggested Patches for Mutation (similar to GGIVLTG)
1	ALLA_ECOLI	GDVIET	33.3	GDVIETQ
2	HCHA_ECOLI	GKLFSTG	42.9	GKLFSTG

**Table 2:** Mutant Library Generated for ALLA_ECOLI and HCHA_ECOLI proteins of *E.coli*. The table shows a list of possible double mutants for wild type proteins and corresponding GRAVY values

**S.No**.	Swissprot ID's	Suggested Patches for Mutation (similar to GGIVLTG)	Patches after mutation	GRAVY value of mutated patch* (Compare with that of GroES mobile loop)**
1	ALLA_ECOLI	GDVIETQ	G**I**VI**I**TQ	1.871
	ALLA_ECOLI	GDVIETQ	G**I**VI**L**TQ	1.771
	ALLA_ECOLI	GDVIETQ	G**I**VI**V**TQ	1.828
	ALLA_ECOLI	GDVIETQ	G**L**VI**I**TQ	1.771
	ALLA_ECOLI	GDVIETQ	G**L**VI**L**TQ	1.671
	ALLA_ECOLI	GDVIETQ	G**L**VI**V**TQ	1.728
	ALLA_ECOLI	GDVIETQ	G**V**VI**I**TQ	1.828
	ALLA_ECOLI	GDVIETQ	G**V**VI**L**TQ	1.728
	ALLA_ECOLI	GDVIETQ	G**V**VI**V**TQ	1.785
2	HCHA_ECOLI	GKLFSTG	G**I**LF**I**TG	2.014
	HCHA_ECOLI	GKLFSTG	G**I**LF**L**TG	1.914
	HCHA_ECOLI	GKLFSTG	G**I**LF**V**TG	1.971
	HCHA_ECOLI	GKLFSTG	G**L**LF**I**TG	1.914
	HCHA_ECOLI	GKLFSTG	G**L**LF**L**TG	1.814
	HCHA_ECOLI	GKLFSTG	G**L**LF**V**TG	1.871
	HCHA_ECOLI	GKLFSTG	G**V**LF**I**TG	1.971
	HCHA_ECOLI	GKLFSTG	G**V**LF**L**TG	1.871
	HCHA_ECOLI	GKLFSTG	G**V**LF**V**TG	1.928

As the single mutations for the patch doesn't make much difference in GRAVY value, so double mutations were considered.

*The GRAVY values are calculated by using ProtParam tool (ExPasy)

**The GRAVY value for mobile loop is = 1.514

### Calculations of aggregation propensity

**Figure 1:**
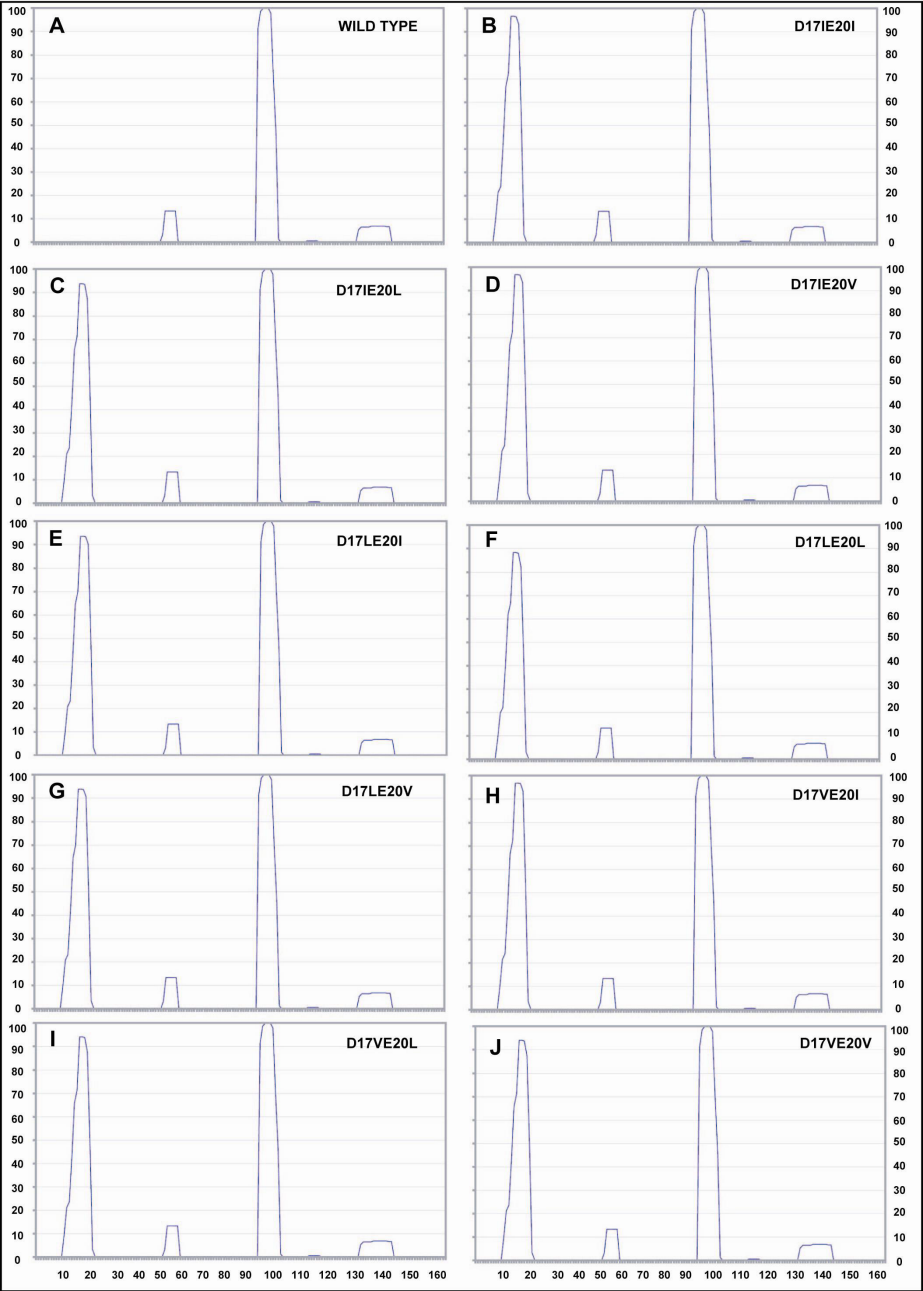
**Aggregation Propensity Plots for ALLA_ECOLI**. The plot shows the aggregation propensity on a scale of 100 and its variation along the amino acid sequence of respective protein. The points corresponding to peaks on graph signifies aggregation prone region on graph. The generation of new peaks or increase in pre-existing peaks can be seen after mutation with hydrophobic residues showing greater propensity to aggregate. (X axis = amino acid residue number; Y-axis = aggregation propensity on scale of 100).

**Figure 2:**
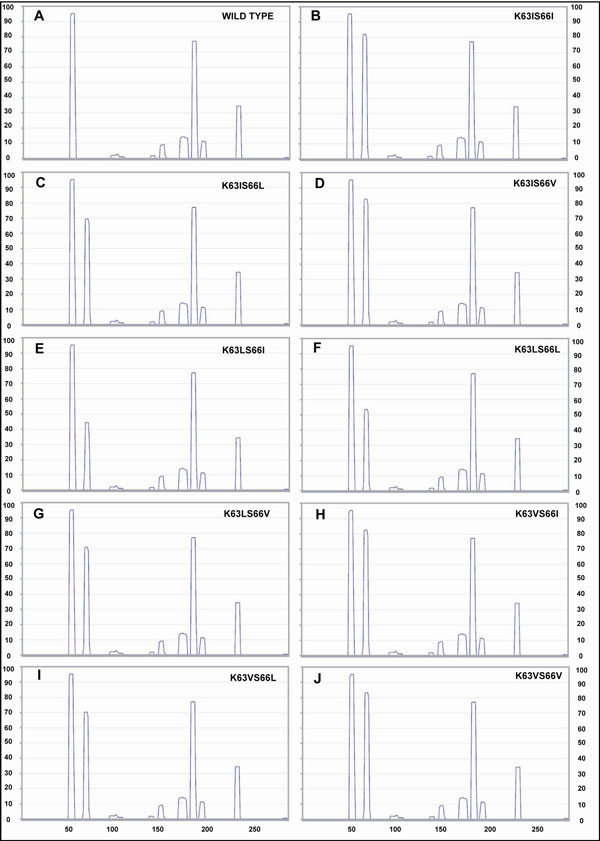
**Aggregation Propensity Plots for HCHA_ECOLI**. The plot shows the aggregation propensity on a scale of 100 and its variation along the amino acid sequence of respective protein. The points corresponding to peaks on graph signifies aggregation prone region on graph. The generation of new peaks or increase in pre-existing peaks can be seen after mutation with hydrophobic residues showing greater propensity to aggregate. (X axis = amino acid residue number; Y-axis = aggregation propensity on scale of 100).

It is known that a protein with greater value of aggregation propensity will have higher tendency to bind with the GroEL [17,18]. We checked the probability of binding between mutants and GroEL by calculating the aggregation propensity of the former under physiological conditions. To check the increase in aggregation propensity of the proteins after mutation, we used TANGO [19-21] and obtained plots of aggregation propensity for these substrates (Figures 1 and 2).

From these plots, we observed that the aggregation propensity of helix and beta sheets of the proteins increases in a certain region of mutants and hence points to an overall effect of decreasing the protein solubility in the physiologic environment.

### Molecular dynamics simulation of the predicted mutants

The generated mutants may or may not be stable at normal physiological conditions. To predict the stability of the mutants, molecular dynamics simulation technique was used [12]. Molecular dynamics (MD) simulation is a form of computer simulation in which atoms and molecules are allowed to interact for a period of time by approximations of known physics, giving a view of the motion of the particles. The technique is based on simple application of Newtonian mechanics at molecular scale. We simulated the conditions under which the behaviour of the macromolecule is to be determined. A force field or potential energy function is applied on various atoms and parts of molecule, and the energy change as function of time is calculated [22-24].

For performing simulations, we used Accelrys Discovery Studio 2.1 with CHARMm as a forcefield. All the computations were performed in windows XP server having Intel Xeon Processor @ 2.93 GHz, with 1.99 GB RAM and was run under SUSE ENTERPRISE LINUX.

### Protein candidates for study

The protein candidates for the current study were chosen by a careful examination of a number of non-substrates [9] of GroEL. Proteins with their known structural data and properties were preferred.

### SwissProt ID: ALLA_ECOLI

This is the SwissProt id for Ureidoglycolate hydrolase found in *E.coli*, which has the PDB ID: 1XSQ[13]. The protein is expressed at 295 K and consists of two chains (both having same sequence of amino acids) in its structure. At the time of expression of protein, the first step is formation of a polypeptide, which then undergoes folding and then formation of the quaternary structure of protein. This suggests that if one considers the binding of GroEL with substrate protein candidates, it does so with the non-native form of the protein i.e. only one chain among two should be considered for the calculation of stability. So for calculating the stability, one should consider the single chain of protein by removing the other chain and polar water molecules from the PDB structure. For the simulation, the Implicit Solvent model Generalized Born with a simple SWitching (GBSW) with dielectric constant equal to 80 was used. Energy minimization was done using Smart Minimizer method with 2000 number of steps. As the method initially calculates the energy of protein at 273 K, the heating step is necessary to calculate the energy at reasonable experimental temperature. Consequently a heating step for finding the energy at 295 K is required.

### SwissProt ID: HCHA_ECOLI

This is the SwissProt ID for Hsp31 protein, a heat shock protein. The PDB ID: 1N57[14]. The protein is expressed at 295 K and consists of two chains (having same sequence of amino acids) in its structure. All the parameters were considered as above, except the temperature range for heating or cooling step. For the heating step, the final temperature was chosen as the temperature at which the protein is expressed i.e. 295 K. The final temperature makes sure for exact mimicking of experimental conditions at which protein is stable.

## Results

For this study, we selected two proteins that have poor binding tendency for GroEL, Ureidoglycolate hydrolase and Hsp31 [13,14]. Our aim was to design several mutants of these proteins and check their physico-chemical parameters like aggregation-propensity, solubility and finally their ability to associate with GroEL. Hydrophobic patches in these proteins that are highly similar with the mobile loop region GGIVLTG in GroES were identified from SIM Alignment tool as shown in Table 1. The change in GRAVY values due to single mutations were not considered substantial and hence double mutants were created for the suggested patches, by replacing the charged amino acid residues with the hydrophobic residues (preferably I, V or L) (Table 2). The GRAVY values for the double mutants were calculated for the obtained patches using Protparam Tool (Expasy) (Table 2). The main behaviour, which we considered with those mutants, was their tendency to aggregate in physiologic conditions, as it has already been shown that the aggregation-prone proteins are more susceptible to bind with GroEL. The stability factors were verified by calculating their energies using MD simulation technique at physiologic conditions. The initial and final (after minimization) energy values for both wild type proteins were calculated, while retaining the same parameters that were employed to calculate the energies of mutants (Tables 3 and 4). Further, the initial and final GRAVY values were calculated by ProtParam for comparison. These observations can be counted for establishing the stabilities of protein mutants.

**Table 3:** Molecular dynamics calculations for ALLA_ECOLI (By using CHARMm force field) Wild type energy calculated from MD simulations = -3214.42774 kcal/mol

**S.No**.	Mutants	GRAVY Value of the patch in wild type	GRAVY value of patch	**Energy calculation from Discovery Studio 2.1 (**kcal/mol)	%age difference from wild type	Increase in GRAVY value
1	D17I E20I	-0.414	1.871	-3140.18459	2.309685	2.285
2	D17I E20L	-0.414	1.771	-3185.526 39	0.899113	2.185
3	D17I E20V	-0.414	1.828	-3193.67046	0.645754	2.242
4	D17L E20I	-0.414	1.771	-3137.01814	2.408192	2.185
5	D17L E20L	-0.414	1.671	-3185.86688	0.888521	2.085
6	D17L E20V	-0.414	1.728	-3170.16685	1.376945	2.142
7	D17V E20I	-0.414	1.828	-3175.30966	1.216953	2.242
8	D17V E20L	-0.414	1.728	-3181.77924	1.015686	2.142
9	D17V E20V	-0.414	1.785	-3170.48382	1.367084	2.199

**Table 4:** Molecular dynamics calculations for HCHA_ECOLI (By using CHARMm force field) Wild type energy calculated from MD simulations = -6038.66825 kcal/mol

**S.No**.	Mutants	GRAVY Value of the patch in wild type	GRAVY value of patch	Energy calculation from Discovery Studio 2.1(kcal/mol)	%age difference from PE of wild type	increase in GRAVY value
1	K63I S66I	0.057	2.014	-5991.49807	0.781135	1.957
2	K63I S66L	0.057	1.914	-6009.53159	0.482501	1.857
3	K63I S66V	0.057	1.971	-6009.52963	0.482534	1.914
4	K63L S66I	0.057	1.914	-6009.52963	0.482534	1.857
5	K63L S66L	0.057	1.814	-6009.52963	0.482534	1.757
6	K63L S66V	0.057	1.871	-5996.11497	0.70468	1.814
7	K63V S66I	0.057	1.971	-5993.80533	0.742927	1.914
8	K63V S66L	0.057	1.871	-6008.70448	0.496198	1.814
9	K63V S66V	0.057	1.928	-6045.64915	-0.1156	1.871

The aggregation propensity considerations were obtained using TANGO plot diagram for each mutant, showing aggregation propensity of amino acids versus their sequence in protein, which shows a change in their behaviour from wild type (Figures 1 and 2).

## Discussion

We have attempted to engineer non-substrate proteins to convert them to the substrates for GroEL. The initial step to this approach was an *in-silico *method for identifying substrate proteins. From a bioinformatics approach, we have identified hydrophobic regions on the non-substrate protein sequences by using an online server, known as SIM alignment tool, in which we got patches similar to that of mobile loop of GroES. The structural similarity to the mobile loop confirms similar interactions with proteins, thereby making them as better candidates. To explore for the increment in their hydrophobic behaviour, all possible permutations of double mutants were considered. The hydrophobic behaviour was measured in terms of GRAVY value, where a greater value of GRAVY signified higher tendency to be insoluble and hence susceptible for aggregation. Keeping this in mind, two hydrophobic amino acids were inserted in place of existing amino acids in the identified patches. Candidates with GRAVY values comparable or greater than that of GroES mobile loop region were selected and compared for their aggregation propensity, to make sure that they act as better substrate under such unfavourable conditions of aggregation, followed by Molecular Dynamics to determine their stabilities. From comparison of aggregation propensity plots, appearance of new peaks or increase in previous peaks could be observed, showing the proposed increase in aggregation propensity of corresponding mutant.

### Selection of candidates

For the selection procedure, a number of mutants were shortlisted, based on their increase in aggregation propensity. In TANGO plots, a new peak was observed due to the addition of hydrophobic amino acid residues. From these selected mutants, we employed another selection procedure to consider the facts of highest GRAVY values and lowest energies. In this way, we identified the following two mutants with comparatively better stability and more aggregation propensity.

D17IE20I from ALLA_ECOLI (energy=-3140.184kcal/mol); (GRAVY = 1.871)

K63IS66I from HCHA_ECOLI (energy=-5991.49807 kcal/mol); (GRAVY = 2.014)

From a careful analysis of the data obtained, we could observe that these two have highest GRAVY values among their family of mutants. Also, it was evident that corresponding energy values and GRAVY values also add up to their increased tendencies to bind with GroEL, where energy value makes sure of their stability on one hand, GRAVY value takes care of aggregation propensity and insolubility.

**Figure 3:**
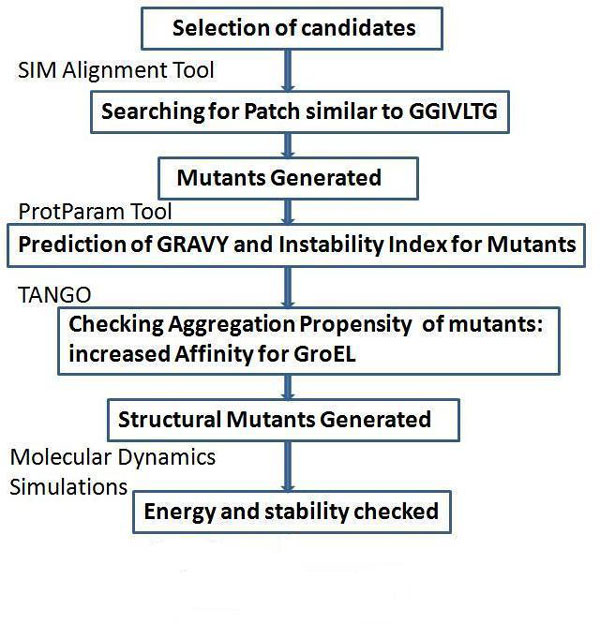
**Scheme for preparation of GroEL substrate**. The scheme shows the logical pathway followed as one moves from selecting protein candidates that are reported as poor substrates of GroEL in a previous study. The hydrophobic patch in the protein sequence, similar to GroES mobile loop region were taken under consideration followed by computational mutation to determine their properties (GRAVY value and aggregation propensity) and energies, which made it possible to select best mutant substrates that can have appreciable binding tendency as well as proper stability.

It has been observed that bacterial chaperonin GroEL and GroES bind newly synthesized or non-native polypeptides through hydrophobic interactions and prevent their aggregation. GroEL and GroES also help in the correct folding of bound substrates. Proteins which bind obligatorily with chaperonin GroEL for the prevention of their aggregation and folding are known as substrates for GroEL. A non-substrate protein is one that does not interact with GroEL, hence even though it is aggregation prone, can't be assisted by GroEL for its folding. We generated mutant protein substrates by an *in silico *approach, which could possibly bind with Chaperonin GroEL with greater affinity as well as with better recognition. This in turn can be folded to its correct native state by using chaperone system with greater efficiency. By performing similar operations on a large number of available protein candidates, one can generate better substrates for Chaperonin GroEL and further, those mutants can be experimentally generated in the future to test their aggregation probability and possibility of chaperone-assisted folding towards functional state. The rationale of the scheme for the preparation of GroEL substrate has been presented schematically in Figure 3.

## Competing interests

The authors declare that they have no competing interests.

## Authors' contributions

VK, AP, DS and TKC designed the methods and experimental setup. VK and AP carried out the implementation of the various methods and drafted the paper. DS and TKC refined the drafted manuscript. All authors have read and approved the final manuscript.
